# The Effect of Curcumin on Postmenopausal Symptoms: A Systematic Review Based on Randomized Controlled Trials

**DOI:** 10.3390/ijms26178260

**Published:** 2025-08-26

**Authors:** Buket Akyakar, İrem Nur Şahin, Duygu Ağagündüz, Dávid Szép, Ferenc Budán

**Affiliations:** 1 Department of Nutrition and Dietetics, Faculty of Health Sciences, Gazi University, Ankara 06490, Turkey; buket.akyakar@gmail.com; 2 Department of Nutrition and Dietetics, Faculty of Health Sciences, İstanbul Beykent University, İstanbul 34398, Turkey; nursahin@beykent.edu.tr; 3 Institute of Physiology, Medical School, University of Pécs, 7624 Pécs, Hungary; szep.david@pte.hu

**Keywords:** curcumin, postmenopausal, symptoms, hormonal balance, phytotherapy

## Abstract

Menopause is a natural and inevitable part of life for women, leading to many physical and psychological changes accompanied by declining estrogen levels. This systematic review aimed to evaluate the effect of curcumin, due to its antioxidant and anti-inflammatory properties, on postmenopausal outcomes in women. This comprehensive analysis of RCTs (randomized controlled trials) published in the last decade was selected through a search of PubMed, Wiley, Scopus, and Web of Science (PROSPERO Identifier: CRD42024549735). Study selection and data extraction were performed using exclusion and inclusion criteria according to the PICOS framework (P: Population, I: Intervention, C: Comparison, O: Outcomes, S: Study designs). Of the twelve studies that met the criteria, 11 had a low-risk bias, but reports were conflicting on serum estradiol levels, bone density markers, and vasomotor symptoms; no significant effects on physical, psychological, or sexual functions were observed. For cardiometabolic biomarkers, short-term curcumin intake showed no significant effects, while long-term interventions using bioavailable forms of curcumin showed improvements in serum fasting glucose, fasting insulin, HOMA-IR (Homeostatic Model Assessment of Insulin Resistance), and lipid parameters. There are a limited number of studies examining the effect of curcumin intake on menopause-related outcomes. While overdose has been observed in some studies attempting to restore estradiol levels, no significant effects have been observed. However, curcumin intake impacts postmenopausal symptoms (e.g., improving symptoms of osteoporosis) through its antioxidant and anti-inflammatory effects. Different forms and doses, combinations, and durations of interventions may influence outcomes. Better-designed studies are needed to understand the potential effects of curcumin intake during menopause.

## 1. Introduction

Menopause is an inevitable part of every woman’s life cycle. The menopausal transition generally occurs between the ages of 45 and 55 and is characterized by the loss of follicular function in the ovaries, leading to decreased estrogen levels in the blood [1]. Estrogen withdrawal can cause many menopause-related effects, including bone loss [2], cardiovascular problems [3], urinary tract dysfunction [4], central nervous system dysregulation [5,6], and vasomotor symptoms [7].

Hormonal and non-hormonal treatments are available for menopausal outcomes [8]. Furthermore, supplementary approaches to treat vasomotor symptoms are currently being discussed and include herbal remedies, behavioral therapy, hypnosis, acupuncture, yoga, and meditation [9]. Indeed, many phytotherapeutic approaches exist to treat gynecological diseases. For example, Aristolochia littoralis, Picralima nitida, Spondias mombin, Sorghum bicolor, and Xylopia aethiopica plants are traditionally utilized in Nigeria for treating dysmenorrhea, and Azeezat et al. recently demonstrated that those plants are not only antioxidant and anti-inflammatory, thus producing analgesic effects, but they can restore the balance of reproductive hormones and improve blood lipid profiles [10].

Various herbal approaches have been discussed for postmenopausal symptoms, including black cohosh (*Actaea racemosa*), red clover (*Trifolium pratense*), kava (*Piper methysticum*), dong quai (*Angelica sinensis*), and evening primrose (*Oenothera biennis*) oil [11]. Dietary intake of curcumin, one of the principal curcuminoid compounds, is also one of the research topics in the regulation of menopausal symptoms [12,13,14]. Curcuminoids—a group of polyphenolic compounds including curcumin, demethoxycurcumin, and bisdemethoxycurcumin—are extracted from the rhizomes of turmeric (Curcuma Longa) and are considered “Generally Recognized as Safe” by the US Food and Drug Administration [15]. While curries contain curcumin, it is also used in various manufactured foods such as mustard sauce, cheese, butter, and chips as a preservative and colorant [16]. There is substantial in vitro and in vivo evidence of antioxidant, anti-inflammatory, antimicrobial, cardioprotective, neuroprotective, anticancer, antirheumatic, and hepatoprotective properties of this yellowish polyphenol [17]. Recent evidence also suggests curcumin’s neuroprotective effects, including the promotion of autophagosome–lysosome fusion in Alzheimer’s disease models [18]. Hence, the potential hormonal regulatory effects of the mentioned plants can pave the way toward advanced phytotherapeutic approaches to treat gynecological diseases if the exact dose–response patterns are elucidated.

Preclinical studies have suggested that curcumin may upregulate 17β-Estradiol (estradiol) levels in the limbic system of ovariectomized rats and modulate the central nervous system [12]. Similarly, while some animal studies have explored its effects on body composition and bone metabolism [14,19,20], these studies involved either multicomponent interventions or in vitro models, which limited their relevance to clinical outcomes [21,22]. Other studies have also modulated signal transduction and oxidative stress in the central nervous system [12,23,24] in postmenopausal women. Curcumin has also been shown to alter gut microbiota diversity in ovariectomized rats in a beneficial manner [14], though the implications for human health remain unclear. In the context of menopause, daily oral hyaluronic acid, chondroitin sulfate, curcumin, and quercetin have been reported to be more effective in preventing urinary tract infections when combined with vaginal estrogen therapy [13]. However, such findings cannot be directly extrapolated to postmenopausal women, and as far as we know, only one human trial has demonstrated a significant effect of curcumin in increasing estradiol levels [25]. The same effect observed from baseline in the placebo group suggests that the hormonal effect of curcumin is questionable. However, the pharmacokinetic properties of curcumin such as poor water solubility, instability at neutral pH, and low oral bioavailability [26] may limit its clinical effects unless administered in specific formulations or in combination with bioavailability enhancers such as piperine, which can increase its bioavailability by up to 20-fold [27].

On the other hand, the beneficial effects of curcumin against menopause symptoms can be reflected in bone health biomarkers, such as total osteocalcin (OCN) and undercarboxylated osteocalcin (unOCN), and Osteopontin (OPN) level. OCN and unOCN are important biomarkers of osteoblast activity and are inversely associated with fasting plasma glucose (FPG) and HbA1c levels [28]. On the other hand, OPN has multifarious effects; it is involved in immune regulation and stress response, cell signaling and tumor progression, as well as bone mineralization and remodeling [29,30,31].

Also, total antioxidant capacity (TAC) is important since a high TAC value is cardioprotective and it is strongly correlated to estrogen levels [32]. The high-sensitivity C-reactive protein (hs-CRP) and malondialdehyde (MDA) represent an inflammatory response and are positively associated with the risk of cardiovascular diseases [33,34].

Glucose-related parameters are important biomarkers, such as serum fasting blood glucose, insulin level, HbA1c level, etc., that are correlated with estrogen level [35]. The same is true for lipid parameters, such as triglycerides, cholesterol, HDL, and LDL levels [35].

Estrogen is also involved in supporting renal and liver function [36]. Thus, biomarkers of renal function, such as serum concentration of Na^+^, K^+^, Cl^−^, HCO_3_^−^, and creatinine, as well as urea nitrogen levels, and glomerular filtration rate (GFR) data were reported during the literature search. Serum levels of aspartate transaminase and alanine transaminase enzymes, bilirubin and albumin levels, and prothrombin time are representative indicators for monitoring liver function.

The literature discusses various health effects of curcumin, including a recent systematic review and meta-analysis of health effects in postmenopausal women [37]. These included studies with heterogeneous designs. Thus, there is a need to conduct a synthesis focused on randomised controlled trials (RCTs) to evaluate the molecular pathways, the relevant biomarkers, and the biological actions of curcumin in postmenopausal women. This systematic review aims to assess curcumin’s benefits, for postmenopausal women from a molecular viewpoint, relying exclusively on evidence from RCTs.

## 2. Methods

This systematic review was registered in the International Prospective Register of Systematic Reviews (PROSPERO) (Identifier: CRD42024549735) and adhered to all the specific guidelines provided by the Preferred Reporting Items for Systematic Reviews and Meta-Analyses (PRISMA) [38]. The research question was formulated as, ‘Does curcumin intake have a positive effect on the health of postmenopausal women?’, which was based on the PICOS framework (Table 1) that stands for Population, Intervention, Comparison, Outcomes, and Study Design. English language studies from the last 10 years were extracted to ensure that this study reflects current and methodologically sound evidence. Two independent researchers (BA and INS) carried out the search, and a third researcher (DA) evaluated the results of the study.

### 2.1. Search Strategy

Web of Science, Wiley, and PubMed databases were used for the literature search. The search keywords were (‘curcumin’ OR ‘curcuma longa’ OR ‘turmeric’) AND (‘menopause’ OR ‘postmenopausal symptoms’ OR ‘bone health’ OR ‘osteoporosis’ OR ‘hot flashes’ OR ‘cardiovascular disease’ OR ‘depression’). The search was restricted to the last 10 years and the English language. In the next step, only randomized controlled trials were selected. The literature research period was from 15 May 2024 to 5 June 2024. The search was re-run on the completion date (1 July 2024), and we found one more study that met the inclusion criteria.

### 2.2. Study Selection and Data Extraction

**Figure 1:**
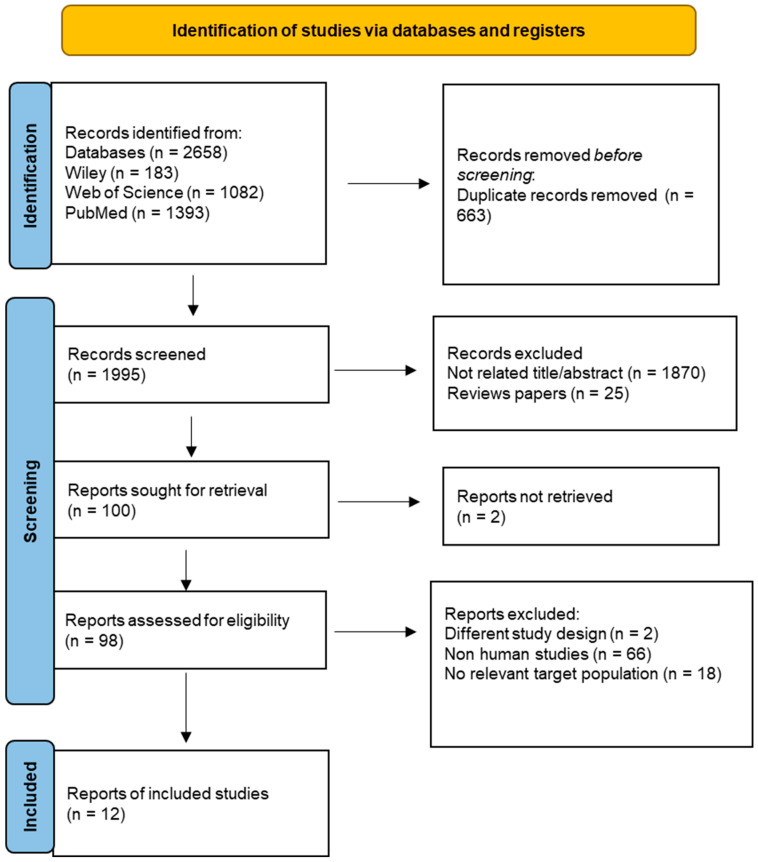
PRISMA flow diagram.

The PRISMA flow diagram of systematic records selection is shown in Figure 1. Two independent researchers (BA and INS) were involved in the literature search to the identification of records that met the inclusion and exclusion criteria. In case of any disagreement, the third researcher (DA) was consulted.

### 2.3. Synthesis Data

The data were presented in Table 2, which includes detailed information about the study’s authors, country, year, population information, intervention strategy, duration, adverse events, and study outcomes.

### 2.4. Evaluation of the Studies’ Quality

The 13-item risk of bias assessment test developed by the Joanna Briggs Institute (JBI) was used to evaluate the quality of randomized controlled trials [50]. 11 out of 12 studies were found to have low risk of bias, while one had a medium risk of bias (Table 3).

## 3. Results

### 3.1. Studies’ Characteristics

Of the 12 studies included, 10 were conducted in Iran [25,39,41,42,43,44,46,47,48,49], including those in health centers in Tabriz [25,42,43,44,47,48,49], and the others in the USA [40] and New Zealand [45]. In addition to postmenopausal women, the study populations also included breast cancer-positive women [25], women with low bone mass density [47], osteopenia or osteoporosis [39,41,42,44,49]. The outcomes were oxidative stress levels, anti-inflammatory parameters, metabolic markers, psychological changes, and vasomotor symptoms.

### 3.2. Curcumin Interventions and Adverse Events

Powdered curcumin, oral capsules (turmeric extract, nanoemulsion, or nanomicelle), and tablets were used, and the duration of the trials ranged from 8 weeks to 12 months. Nanoformulations (nanomicelles or nanoemulsions) are known to increase solubility and absorption, which may lead to increased therapeutic efficacy compared to powder or tablet forms [51]. Except for one experiment with bioactive yoghurt with curcumin, the duration was a minimum of 1 week [45]. Although some trials did not report any side effects [45,49], the most common side effects were gastrointestinal complaints such as nausea, vomiting, belching, diarrhea, abdominal pain, constipation, dyspepsia, and unpleasant taste reported in other trials [25,39,40,42,43,44,46,47,48]. For the sake of completeness, it should be mentioned that the greatest dose of daily curcumin intake in a human clinical study was 12,000 mg, and for the patients, the bulky volume was not acceptable, and up to 8000 mg/day, there were no toxic effects observed [52].

### 3.3. Serum Estradiol Levels

One study with a 3-month nanomicelle curcumin intervention, part of aromatase inhibitor therapy, for postmenopausal women with breast cancer found no change in serum estradiol levels compared to the placebo group [40]. A 6-month intervention in healthy postmenopausal women observed within-group increases in serum estradiol levels in participants receiving nanomicelle curcumin capsules or nanomicelle curcumin capsules combined with Nigella sativa oil compared to baseline [25]. However, there was no statistically significant difference between the treated and placebo groups [25]. Therefore, current evidence from human studies suggests that curcumin does not significantly alter the serum estradiol levels in postmenopausal women compared to placebo [25] (nanomicelle curcumin capsule), [40] (nanoemulsion curcumin).

### 3.4. Curcumin and Oxidative Stress and Inflammation During Menopause

Regarding oxidative stress and anti-inflammatory biomarkers, in an 8-week study, postmenopausal women who received 500 mg curcumin (turmeric root extract containing 95% curcuminoids) twice daily showed significantly lower mean serum MDA levels [MD (95% CI): −0.5 nmol/mL (−0.8 to −0.1); *p* = 0.009] and hs-CRP levels [MD (95% CI): −0.5 mg/L (−0.8 to −0.2); *p* = 0.025], while their TAC levels increased [MD (95% CI): 0.2 mmol/L (0.1 to 0.35); *p* < 0.001] compared to the placebo group [43]. However, the study also stated that these differences were not statistically significant when compared with the placebo group. Therefore, the results do not support a definitive effect of curcumin on oxidative stress or inflammation in postmenopausal women. Another study observed that participants in a 6-month trial receiving 80 mg/day nanomicelle curcumin had higher serum SOD and TAC levels than the placebo group (*p* = 0.013 and *p* = 0.011, respectively); though no significant changes were observed in MDA levels [42]. In parallel, another study showed there was no association for MDA levels; the curcumin (turmeric root extract containing 95% curcuminoids) and ginger co-supplementation group showed a significant increase in TAC levels versus placebo, whilst either curcumin (turmeric root extract containing 95% curcuminoids) or curcumin plus ginger increased the serum SOD levels [41]. There were no significant decreases of serum cytokines, including tumor necrosis factor-alpha (TNF-α) and interleukin-6 (IL-6) [41].

### 3.5. Curcumin-Adiposity and Metabolic Changes During Menopause

There was no significant difference in 30 min, and 1, 2, 3, and 4 h postprandial serum triglycerides, insulin, and glucose levels between the curcumin-intervention group (who received bioactive yoghurt with 103 mg curcumin) and the placebo group in a crossover randomized controlled trial [45]. Postmenopausal women receiving 500 mg curcumin twice daily for 8 weeks had significantly lower serum fasting blood glucose, total cholesterol, and triglyceride levels compared to the placebo group [46]. There was a significant decrease in plasma fasting insulin and HOMA-IR levels in both nanomicelle curcumin capsule and co-supplementation (nanomicelle curcumin capsule + Nigella sativa) versus placebo with a lower amount but higher bioactive form (80 mg/day nanomicelle curcumin) [25]. Moreover, despite the differences in curcumin formulations and dosages, two investigations revealed that there was no significant difference, when comparing the group receiving curcumin to the placebo group, in the results of renal and hepatic function tests [44] (nanomicelle capsule), [46] (Turmeric root extract containing 95% curcuminoids).

### 3.6. Curcumin and Bone Health During Menopause

Curcumin inhibits the genesis of osteoclasts and improves BMD and bone strength by reducing the production of nitric oxide and free radicals, inhibiting the synthesis of inflammatory cytokines, and inhibiting nuclear factor-kappa B (NF-κB) [49,53]. The results from curcumin and its formulations intake and bone density indices; the total hip bone mineral density, the femoral neck bone mineral density, and the total body bone mineral density were higher in the Alendronate + nanomicelle curcumin group after a 12-month intervention, compared to the control [49], and the T-score and BMD in the femoral area of nanomicelle curcumin and nanomicelle curcumin + *Nigella sativa* groups were significantly higher; and significantly increased the T-score and BMD in lumbar-spine area for the nanomicelle curcumin + *Nigella sativa* group [39]. In contrast, there were no significant differences in femoral neck and lumbar spine bone mineral density between the intervention groups [curcumin: turmeric extract (52.21 ± 5.17 mg) + turmeric powder (468.84 ± 18.22 mg) or curcumin (same formulation) + ginger ] and placebo [41]. In terms of upper extremity functions and pain, Lustberg et al. [40] showed that consumption of curcumin (nanoemulsion) for three months had no effect on shoulder, arm, and hand function and joint symptoms in breast cancer patients who were postmenopausal and experiencing joint discomfort as a result of continuing adjuvant aromatase inhibitor medication. Although some positive effects on bone mineral density have been observed with nanomicelle curcumin formulations, particularly those combined with Alendronate or *Nigella sativa*, the heterogeneity in curcumin formulations, dosages, and intervention durations among the included treatments makes it difficult to draw definitive conclusions about its efficacy.

One study showed that an increase in microRNA-21 (miRNA-21) expression levels was observed in the combination of nanomicelle curcumin with Nigella sativa, while there was no difference for miRNA-422 and miRNA-503 expression levels in postmenopausal women with poor bone mineral density [47]. Studies are contradictory regarding the increase or decrease in miRNA-21 levels in patients with osteoporosis in comparison to healthy people [47]. Among postmenopausal women, miR-21 expression level in the serum and plasma of women with osteopenia or osteoporosis was significantly reduced in comparison to the women with normal bone density [47]. Considering the studies we included, which analyzed serum indicators of bone turnover, ginger and curcumin (turmeric root extract containing 95% curcuminoids) intake decreased serum OCN and alkaline phosphatase levels among postmenopausal women with primary osteoporosis [41]. However, other co-supplementations such as Nigella sativa oil and nanomicelle curcumin or Alendronate and nanomicelle curcumin had a significant decline in alkaline phosphatase levels [46], but not OCN or OPN levels [44,49]. In the study of Kheiridoost et al., there was a significant increase in serum OCN and OPN levels in nanomicelle curcumin-treated groups compared to the baseline, and an increase in the OPN level in the nanomicelle curcumin-treated groups [44]. Nigella sativa oil + nanomicelle curcumin-treated group. One trial showed that the Alendronate + curcumin intake significantly reduced the mean serum level of type I collagen’s C-terminal cross-linking telopeptide compared to the control group, referring to decreased bone turnover. Also, in the group receiving 110 mg curcumin combined with alendronate, BALP and CTx levels showed a significant decrease, while OCN levels significantly increased by the end of the study compared to both the control and alendronate-only groups [49]. OPN gene expression is activated by inflammatory signal transducers, such as IL-1, TNF-α, and PDGF, through the activation of protein kinase C [54]. Thus, theoretically, the anti-inflammatory effects of curcumin hindering the mentioned signal transducers may inhibit OPN expression [55].

### 3.7. Curcumin, Psychological Outcomes and Vasomotor Symptoms During Menopause

Some studies of the systematic review showed that an 8-week curcumin (standardized turmeric root extract, 95% curcuminoids) intervention had no significant effect on postmenopausal symptoms including psychological, physical, and vasomotor symptoms and sexual dysfunction, while one study with a 6-month nanomicelle curcumin intervention found improvements only in vasomotor symptoms, both compared to the placebo group [39,43,48] (turmeric root extract containing 95% curcuminoids).

At an 8-week intake of 1 g of curcumin per day, one study showed that curcumin (standardized turmeric root extract, 95% curcuminoids) consumption had a significantly lower frequency of hot flashes compared to placebo [48], while another study found no association [46].

## 4. Discussion

This systematic review focused on synthesizing the available data on the possible benefits of curcumin intake in the postmenopausal stage. This review aimed to understand the effect of curcumin intervention on postmenopausal symptoms and outcomes; therefore, only randomized controlled trials were evaluated.

There were conflicting reports in the literature on the effect of curcumin supplementation on menopausal estrogen deficiency. One preclinical study reported a significant increase in serum estradiol levels and observed an increase in aromatase mRNA expression in the limbic system in ovariectomized Wistar female albino rats receiving 100 mg/kg/day curcumin compared to the control group [12]. However, Zhang et al. [14] showed that 12 weeks of 100 mg/kg daily curcumin intake by gavage did not increase the low estradiol levels in ovariectomized Wistar rats. In parallel with these results, no significant changes in serum estradiol levels compared to placebo were observed in this systematic review. Accordingly, although the intake of curcumin and its metabolites up-regulated low estrogen levels in ovariectomized rats, no positive effect was observed in human studies. This can be explained by the results of Lu et al., who demonstrated that curcumin (IC50, 3.49 μM) (and most of its derivatives, too) is a potent aromatase (CYP19A1 enzyme) inhibitor, thereby hindering the synthesis of estrogens [56].

It has been reported that menopause-like changes in rats with surgically removed ovaries affect oxidative stress parameters [20]. Curcumin—the primary bioactive compound among a group of curcuminoids found in turmeric—plays an important role in oxidative stress and anti-inflammatory properties via certain pathways [17]. This polyphenolic compound downregulated liver lipoperoxidation and modulated non-protein and protein sulfhydryl content in supplemented rats [20]. However, the results of the present study were conflicting concerning antioxidant capacity and inflammatory markers of curcumin intake. The use of different curcumin formulations (powder, extract, nanoemulsion, nanomicelle), combinations (e.g., standard treatment, *Nigella sativa*, ginger), varying intervention durations, and diverse study populations may have affected these results.

It has been hypothesized that curcumin and its metabolites may exert beneficial effects on adipose tissue distribution and metabolic changes associated with menopause. In ovariectomized rats receiving 100 mg/kg/day curcumin treatment, greater lean body mass, greater lean mass/fat mass ratio, and less intestinal adipose tissue accumulation were reported compared to the control group [19,20]. It also reduced weight gain resulting from estrogen deficiency [14]. Curcumin induces peroxisome proliferator-activated receptor-γ (PPAR-γ), which modulates LDL receptors, leading to the amelioration of hypercholesterolemia [57]. In addition, ovariectomized rats receiving 100 mg/kg/day, curcumin revealed a noteworthy reduction in serum leptin levels and revealed a noteworthy reduction in mRNA levels of Lpl and Cd36, genes associated with fatty acid uptake, and a significant increase in adiponectin/leptin ratio [19]. We did not observe significant differences in metabolic markers for short-term curcumin intervention, while for longer-term and higher bioactive strategies, we found significant changes in serum fasting blood glucose, total cholesterol, triglyceride, plasma fasting insulin, and HOMA-IR levels. Long-term use of curcumin may contribute favorably to glucose and lipid profiles in the postmenopausal period. Consistently, Morrona et al. [20] found no significant differences between the group treated with 100 mg/kg/day curcumin and the sham-operated group, in serum ALT and AST levels. In parallel, no significant effect of curcumin intake on liver function was found in the present study.

Osteoporosis, one of the most important health problems of menopause, is a metabolic bone disorder, and its major factor is low estrogen levels in postmenopausal women [47]. Menopause-related estrogen depletion upregulates RANKL production, which increases the number of osteoclasts and activates them, leading to the suppression of osteoblasts [58]. Curcumin has been reported to attenuate oxidative stress-induced apoptosis in osteoblasts by protecting mitochondrial activities and enhancing phosphorylated protein kinase B and phosphorylated glycogen synthase kinase-3β levels [21]. It was also found that curcumin is a potential activator of autophagy in osteoclast precursors [22]. In an experimental study, El-Nasr et al. [59] found that after a two-month intervention with curcumin plus fenugreek, the femur, tibia, and total bone mineral density of the ovariectomized Wistar rats increased, and only curcumin intake decreased the bone loss (Curcumin from Curcuma longa, 65% purity, formulated in PLGA nanoparticles). Moreover, the poly(lactic-co-glycolic acid)-conjugated form of curcumin showed a better effect on osteoporotic bone loss of ovariectomized Sprague-Dawley rats compared with free curcumin [60]. The contribution of curcumin to miRNA-21 expression can be relevant since miRNA-21 promotes osteogenic differentiation and mineralization in MC3T3-E1 cells by enhancing the expression of key osteogenic markers, including OPN, ALP, Runx2, Osterix, and Mef2c. Conversely, miRNA-21 deficiency leads to a reduction in the expression of these markers at both the mRNA and protein levels, highlighting its positive regulatory effect on osteoblast differentiation [61]. Estrogen increases osteoclast apoptosis by downregulating miRNA-21 biogenesis. miRNA-21 suppresses the inhibitory effect of the c-Fos factor on the RANKL-induced process of osteoclast genesis by decreasing the level of programmed cell death protein, which leads to increased osteogenesis [47]. Thus, in this context, Nigella sativa and curcumin in combination could mimic the anti-osteoporotic effects of estrogen. In the results of this study, curcumin alone did not have a significant positive effect on bone health, but potential positive effects were observed with existing medical treatment or in combination with other herbs (e.g., Nigella sativa). This reinforces the idea that curcumin may be a potential complementary agent for menopausal bone health.

A systematic review showed that miRNAs are potential biomarkers for the diagnosis and prognosis of osteoporosis [62]. An experimental study reported that 110 mg/kg/day curcumin for 12 weeks downregulated EZH2 mRNA levels but upregulated β-catenin and Runx2 in the mandible and femur of ovariectomized osteoporotic rats [63]. However, this systematic review found that curcumin alone had no considerable effect on miRNA expression.

It is thought that estrogen deficiency during menopause may affect mental health [64]. Animal models show that in Wistar female albino rats following ovariectomy, there was activation of monoamine oxidase A, a catabolic enzyme, while at the same time, there was a significant decline in 5-HT (serotonin) anabolic factors [12]. Conversely, curcumin and its bioactive derivatives are believed to have advantageous properties for postmenopausal anxiety-like behaviors and depression by reducing neuroinflammation and providing neuromodulation. In ovariectomized rats receiving 100 mg/kg/day of curcumin, immobility in the forced swim test was significantly reduced, and the duration of struggle was prolonged versus the control group [12]. Moreover, 100 mg/kg/day curcumin intake improved their performance in the open field test and the elevated plus maze test [23,24]. Curcumin intake increased serotonin (5-HT) levels in various brain areas and plasma compared to the control group [12], and it altered the levels of dopamine and norepinephrine in different parts of the brain [24]. A significant increase in 5-HT1A (5-hydroxytryptamine1A) and Tryptophan hydroxylase-2 (TPH-2) mRNA expression, and a notable decline in the mRNA expression of monoamine oxidase, a catabolic enzyme, were observed in the rat group receiving 100 mg/kg curcumin daily [12,24]. Curcumin (100 mg/kg/day) intake increased brain-derived neurotrophic factor (BDNF) mRNA expression and ERK1/2 protein levels in the limbic system [12].

In experimental studies, it was thought that curcumin (50 and 100 mg/kg/day) is a potential modulator of oxidative stress and inflammatory pathways in the central nervous system [23,24,65]. It was observed that ovariectomized Wistar rats receiving daily 50 mg and 100 mg/kg curcumin intake considerably decreased lipid peroxidation in the frontal cortex and striatum, increased catalase and glutathione peroxidase actions in the frontal cortex and hippocampus, reduced oxidative damage, and improved anxiety-like behaviors [23]. In addition to reducing oxidative-nitrosative stress in the central nervous system, curcumin has been documented to prevent neuroinflammation by reducing serum corticosterone hormone, TNF- α, IL-β1, and IL-6 levels in the limbic system of ovariectomized rats [24]. However, according to our study findings, curcumin (in various forms including powdered curcumin, such as oral capsules and turmeric extract, nanoemulsion, nanomicelle, and tablets) did not show significant effects on physical, psychological, and sexual functions in the postmenopausal period, and contradictory reports were observed in hot flashes and vasomotor symptoms.

**Figure 2:**
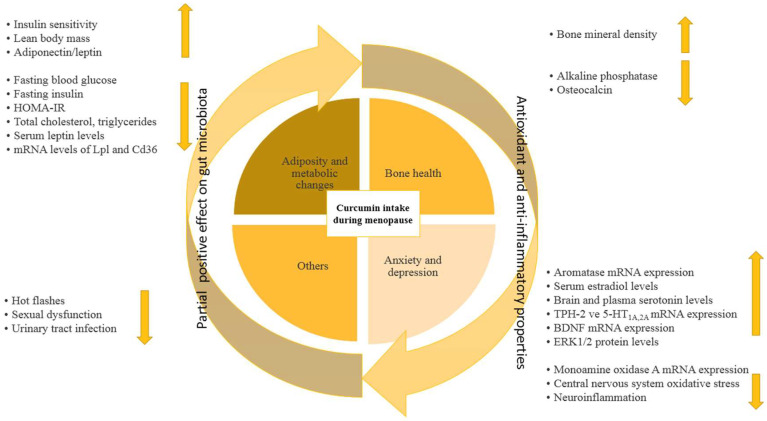
Proposed beneficial effects of curcumin intake during menopause. The central circle shows curcumin consumption, which affects four major domains: adiposity and metabolic changes, bone health, anxiety and depression, and other menopause-related symptoms. Curcumin exerts its effects through antioxidant, anti-inflammatory, and the partial gut microbiota modulation pathways (as indicated by the arrows). Upward arrows project an increase in relevant markers or outcomes; downward arrows represent a reduction.

Even though it is claimed that curcumin and its bioactive forms have promising effects for postmenopausal women, their mechanisms are still unclear. The studies generally emphasized the synergistic effect of curcumin, and considered it a complementary therapy rather than a substitute for medical treatment [13,44,49]. This synergistic effect may be considered to contribute to the current medical treatment or other herbal therapies due to the anti-inflammatory and antioxidant effects of curcumin. In general, the proposed potential effects of curcumin (in multiple forms, including powder, oral turmeric extract capsules, nano-, and micro-formulations, and tablet intake) on postmenopausal outcomes are shown in Figure 2. Although animal experiments have yielded promising results [12,19,20,23,24], randomized controlled trials with either a curcumin-only intervention or dose–response designs are needed to understand how curcumin affects menopausal outcomes.

This review includes trials using various turmeric derivative preparations, including curcumin, curcuminoids, and turmeric powder/extracts. These were administered in many different varieties, including 80 mg nanomicelle capsules, turmeric extract capsules containing 475 mg curcuminoids, a mixture of turmeric powder and extracts, and nanoemulsion formulations. Doses ranged from 80 mg to 1000 mg daily, with treatment durations from eight weeks to 12 months. The European Food Safety Authority (EFSA) has determined the safe quantity of curcumin at 140 mg/day, but reports an adult’s daily maximum intake as 300 mg (excluding pregnancy and lactation) [66]. On the other hand, turmeric powder contains an average of 3% curcuminoids [17]. Considering that 1 to 1.5 teaspoons of turmeric powder contains about 3 to 4.5 g [67], this amount contains about 90–135 mg of curcuminoids. This is the established safe daily dose. However, turmeric extracts containing 95% curcuminoids, provide an average of 475 mg curcuminoids per 500 mg oral capsule in some trials of this systematic review [43,46]. Although doses of 500 to 1200 mg of turmeric powder extract have been reported to be safe in healthy people [68], its use during menopause is still uncertain. This overdosage is clearly evident in the studies included in this systematic review. Moreover, some side effects were reported with turmeric extract or its bioactive forms. Even at these doses, the positive effect of curcumin intake on menopausal outcomes is still questionable.

On the other hand, curcumin, as its active metabolites also do, exerts significant anti-inflammatory, immunomodulatory, and anticancer effects through modulating intracellular signal transduction mechanisms. Curcumin and related bioactive compounds inhibit inflammation by suppressing inducible nitric oxide synthase (iNOS), chemokine receptor type 4 (CXCR-4), CRP, inflammatory matrix metalloproteases (MMPs), AP-1, NF-κB, TNF, LOX, COX2, 5-LOX, HER2, EGFR, IL-1β, IL-6, IL-23, PGE2, as well as inhibiting STAT3 phosphorylation, BCL2, cyclin D1, relevant in carcinogenesis [69,70,71,72,73]. Furthermore, curcumin and its derivatives increase glutathione-s-transferase enzyme expression, protein levels, as well as glutathione levels [69,74]. Many factors regulate DNA methylation activity and facilitate DNA methyltransferase (DNMT) recruitment to DNA in synchrony with histone deacetylase (HDAC) activity [75]. Curcumin and its metabolic derivatives inhibited DNMT1 elevated by mTOR in vitro, presumably while curcumin and its metabolic derivatives upregulate lncRNA NBR2, which is an inhibitor of the Akt/mTOR pathway and ultimately induces apoptosis [76,77,78]. Since the mentioned signal transducers are mostly involved in the discussed postmenopausal symptoms and outcomes, the beneficial effects of curcumin regarding postmenopausal symptoms could be considered as “a resultant of several coincidental molecular biological effects” rather than “estrogen supplementing or mimicking effect”.

There are some strengths and limitations in the study. This is the first systematic review to examine the effect of curcumin consumption on menopause-related outcomes by including only RCTs. It is also predicted that the intake of curcumin and its analogues on menopausal complaints will shed light on future studies. The heterogeneity of the interventions (location, population differences, various doses and forms of curcumin intervention, and duration of intervention) complicated the interpretation of the trials. Furthermore, combination interventions obscured the main effect of curcumin and chemically related substances. In particular, in some of the trials, differences between the intervention and control groups (e.g., age, BMI, and physical activity) may have influenced the results of the research. In addition, there was no information about the effect of loss to follow-up on the results of the analyses or whether the groups were analyzed as randomized, which affects study bias as well. To comprehend how curcumin and its forms affect postmenopausal outcomes, these issues must be addressed in the design of future studies.

## 5. Conclusions

This systematic review aimed to examine the potential effects of curcumin supplementation during postmenopausal period. Although experimental studies show promise that curcumin intake may have favorable effects on postmenopausal outcomes, there are conflicting and limited results in RCTs. Different curcumin forms/dosages and combinations of interventions and different durations of interventions may influence the results. At present, the efficacy of curcumin in managing menopause-related symptoms and biomarkers cannot be conclusively established. However, many in vitro experiments suggest significant anti-inflammatory effects of curcumin enabling to treat postmenopausal symptoms through modulating intracellular and extracellular signal transduction processes. Well-designed, placebo-controlled trials are needed to understand the role of curcumin intake during menopause.

## Figures and Tables

**Table 1:** The PICOS framework.

Parameter	Inclusion Criteria	Exclusion Criteria
P (Population)	Inclusion: (1) Healthy postmenopausal women, (2) Postmenopausal women with postmenopausal outcomes (e.g., low bone mass density, hot flashes, adiposity), (3) Postmenopausal women receiving treatment for the symptoms/diseases (e.g., depression, osteoporosis, cancer).	Women in the perimenopausal age
I (Intervention)	Intervention: (1) Curcumin, (2) Curcumin + Vitamin D/Vitamin E/Calcium or other supplements used for the symptoms of the postmenopausal term (3) Curcumin + other phytochemicals (4) Curcumin + herbal therapies (5) Curcumin + medical treatment	-
C (Comparison)	Comparator: Control, Placebo (1) Healthy postmenopausal women (2) Postmenopausal women with postmenopausal outcomes (e.g., low bone mass density, hot flashes, adiposity) (3) Postmenopausal women receiving treatment for the symptoms/diseases (e.g., depression, osteoporosis, cancer)	Other comparisons
O (Outcomes)	Serum estrogen, aromatase enzyme levels, and various gene expressions will be evaluated for the monitoring of menopausal symptoms and diseases. Serum parameters such as OCN, OPN, bone-specific alkaline phosphatase, and C-terminal cross-linking telopeptide of type I collagen for bone health/bone turnover markers; Dual Energy X-ray Absorptiometry (DEXA) measurement results for bone mineral density, valid and reliable pain scales used for bone strength or bone/joint pain. Serum superoxidase dismutase (SOD), TAC, MDA for oxidative stress parameters; serum CRP (or hs-CRP, respectively, ILs, TNF- α to evaluate inflammatory response. The number of hot flashes in a week, valid and reliable scales for anxiety/depression evaluation. Glucose and lipid parameters such as serum fasting blood glucose, HbA1c, insulin, triglycerides, cholesterol, HDL, and LDL levels. For adiposity, the waist circumference and hip circumference ratio, bioelectrical impedance analysis for body analysis, and body mass index (BMI). For evaluating renal function: Serum Na^+^, K^+^, Cl^−^, HCO_3_^−^, creatinine and urea nitrogen levels, glomerular filtration rate (GFR). For liver function evaluation: Serum levels of aspartate transaminase, alanine transaminase, bilirubin, albumin, prothrombin time	Other measurements
S (Study designs)	Randomized controlled trials	Cross-sectional studies, longitudinal studies, cohort studies, case–control studies, systematic review, narrative review, animal experiments, case reports, abstracts, letters to the editor

**Table 2:** Characteristics of Included Studies.

Author, Year, Country	Study Design	Population	Intervention		Duration	Adverse Events	Study Outcomes
			Intervention	Control			
Usefian et al., 2024 [39]	Placebo-controlled trial Factorial RCT-Triple-blind	Postmenopausal women diagnosed with primary osteoporosis or osteopenia. Mean menopausal age: 48.3 (3.9) (*n* = 120)	Intervention 1: 80 mg/day curcumin (nanomicelle) (*n* = 30) Intervention 2: 1000 mg *Nigella sativa* oil + 80 mg/day curcumin (nanomicelle) (*n* = 28) All participants received alendronate 70 mg tablets once a week-500 mg calcium + 400 units vitamin D daily.	Placebo (*n* = 29)	6 months	Nausea, belching, bad taste, reflux, cough, drowsiness, bloating, stomach pain, and dry mouth (in both interventions), headache (in Intervention 2)	There were no significant changes among the groups for the bone density indices (*p* > 0.05). In both intervention groups there were significant improvements in the mean overall quality of life score in contrast to the baseline, but no discernible change between the groups. The vasomotor dimension in the Intervention 1 group was significantly better in the post-intervention comparison than in the Placebo group; adjusted difference (95% CI): −0.90 (−1.62 to −0.18), *p* = 0.014. There were no remarkable changes among groups in other dimensions.
Lustberg et al., 2024 [40]	RCT-Double blind	Postmenopausal women with stage I-IIIa estrogen receptor—and/or Progesterone receptor-positive breast cancer whose joint pain worsened or lasted longer than three months as a result of continued adjuvant aromatase inhibitor medication. Median age: 60 (39–72) (*n* = 34)	200 mg/day curcumin (nanoemulsion) (*n* = 19)	Placebo (*n* = 15)	3 months	Grade 1: symptoms include flatulence, diarrhea, nausea, heartburn, headaches, constipation, dyspepsia, and belching. Grade 2: headache, heartburn, and dyspepsia	There was no significant change in the scores of the Functional Assessment of Cancer Treatment-Endocrine Symptoms, Disabilities of the shoulder, arm, and hand, and the Brief Pain Inventory—short form in the intervention group. Grip strength of the right arm had risen significantly >2 kg in the intervention group compared to placebo (*p* = 0.05). In the intervention group, no change for serum estrone and estradiol levels was observed.
Salekzamani et al., 2023 [41]	RCT-Triple blind	Women who were postmenopausal and have been diagnosed with primary osteoporosis (*n* = 120)	Intervention 1: 2 curcumin tablets [turmeric extract (52.21 ± 5.17 mg) + turmeric powder (468.84 ± 18.22 mg)]/day + 2 ginger placebo/day (*n* = 30) Intervention 2: 2 ginger tablets (30.44 ± 4.11 mg) + 2 curcumin tablets [turmeric extract (52.21 ± 5.17 mg) + turmeric powder (468.84 ± 18.22 mg)] /day (*n* = 27) All participants received 70 mg alendronate weekly + daily calcium (500–1000 mg) and vitamin D3 (600–800 IU)	Placebo (*n* = 30)	4 months	No crucial adverse event	There were significant increases in femur neck bone mineral density in Intervention 1 mean changes (MC) (MC = 0.07 [95% CI: 0.04–0.10]), and Intervention 2 (MC = 0.05 [95% CI: 0.02–0.09]), compared to the baseline level. No significant differences were found between groups in femoral neck and lumbar spine bone mineral density when adjusted for baseline measures, BMI, and age. There were significant decreases in serum OCN level, the changes were in Intervention 1 [MC = −2.65 (95% CI: −5.82 to −1.05)] and Intervention 2 (MC = −4.22 [95% CI: −7.98 to −1.54]) The intervention 2 group was more effective at decreasing the serum OCN level (*p* = 0.019) compared to placebo. Between-group analysis revealed a significant difference between the intervention group 2 and the placebo group (*p* = 0.038). After adjusting for baseline values, age, BMI, and femoral neck BMD, the ginger plus curcumin treatment was more effective than placebo in reducing serum OCN levels (*p* = 0.019). There was a significant decline in serum ALP in Intervention 2 (MC = −36.89 [−63.55 to −10.23]). After adjustment for baseline measurements, including BMI, age, and femoral neck bone mineral density (BMD), Intervention 2 was more effective at reducing serum ALP levels compared to placebo (*p* = 0.007). No significant changes for TNF-α between groups were observed, except in the intervention 2 group, where TNF-α decreased significantly compared to the baseline value (MC = −10.86 [95% CI: −12.73 to −0.02]) (*p* = Not defined). According to between-group analysis, there were significant differences between Intervention 1 versus placebo (MC = −0.34 [95% CI: −0.53 to −0.16]) and Intervention 2 versus placebo groups (MC = −0.41 [95% CI: −0.64 to −0.25]) in the decline of serum hs-CRP levels (*p* = 0.035 and *p* = 0.018, respectively). After adjusting for baseline values, BMI, and age, it was found that intervention 2 was significantly more effective than placebo in lowering serum hs-CRP levels (*p* = 0.042). Serum hs-CRP concentrations decreased significantly in Intervention Group 1 (MC = −0.34 [95% CI: −0.53 to −0.16]) and Intervention Group 2 (MC = −0.41 [95% CI: −0.64 to −0.25]). The between-group analysis showed a significant decrease in the serum levels of hs-CRP for both interventions compared to the placebo. In the case of intervention 1 group data, a significant decrease was observed compared to the placebo, as well as in the case of intervention 2 group compared to the placebo group (*p* = 0.035 and *p* = 0.018, respectively) in the serum levels of hs-CRP was observed. There was no significant variation in IL-6 between groups, even after adjusting for baseline values, BMI, and age. However, in both interventions IL-6 showed a decreasing trendline, while in the placebo group it increased. There was a significant difference in Intervention 2 (MC = 1.19 [95% CI: 0.97–2.41]). Intervention 2 was more effective at increasing serum TAC levels compared to placebo (*p* = 0.012). No significant variation for serum MDA level between groups was presented The Intervention 1 and Intervention 2 groups showed a significant increase in serum SOD levels, compared to placebo (*p* = 0.010 and *p* = 0.003, respectively).
Sadeghzadeh et al., 2023 [25]	RCT-Triple-blind	Postmenopausal women, mean age: 58.4 (3.7) (*n* = 120)	Intervention 1: 80 mg/day curcumin (nanomicelle capsule) (*n* = 30) Intervention 2: 1000 mg *Nigella sativa* oil + 80 mg/day curcumin (nanomicelle capsule) (*n* = 28)	Placebo (*n* = 29)	6 months	Nausea, belching, headache and unpleasant taste (in both groups)	There was a significant decrease in FI levels in Intervention 1 and Intervention 2, versus placebo (*p* = 0.001 for both) A significant decrease of HOMA-IR levels in Intervention 1 and Intervention 2 was observed compared to placebo (*p* = 0.001 for both). No significant difference between Intervention groups in FBS, TG, TC, HDL, LDL, and mean serum estradiol levels In the Intervention 1 group, TC and LDL levels decreased while HDL and estradiol levels increased after intervention (*p* = 0.001, *p* = 0.08, *p* = 0.08, and *p* = 0.05, respectively) In the Intervention 2 group, TG and TC levels decreased while HDL and estradiol levels increased after intervention (*p* = 0.05, *p* = 0.001, *p* = 0.001, and *p* = 0.013, respectively)
Iranshahi et al., 2023 [42]	Factorial RCT-Triple-blind	Postmenopausal women with primary osteoporosis or osteopenia, mean age: 57.79 ± 3.76 (*n* = 115)	Intervention 1: 80 mg/day curcumin (nanomicelle capsule) (*n* = 30) Intervention 2: 1000 mg *Nigella sativa* oil + 80 mg/day curcumin (nanomicelle capsule) (*n* = 28)	Placebo (*n* = 29)	6 months	Mild nausea, vomiting, belching, headache, and unpleasant taste sensation in the beginning of the study	There were significant increases in the SOD serum levels of Intervention 1 and Intervention 2 groups, and the value of the SOD serum levels posttest in the Intervention 2 group was significantly higher than the placebo (MD = 100.4, 95% CI = 21.9–178.9, *p* = 0.013). In the Intervention 2 group, TAC serum level significantly increased. The value of its posttest in this group was significantly higher than the placebo group (MD = 0.23; 95% CI = 0.05–0.41; *p* = 0.011). No significant difference in MDA serum levels was observed between the groups
Farshbaf-Khalili et al., 2022 [43]	RCT-Triple-blind	Postmenopausal women, mean age: 52.3 ± 2.9, (*n* = 84)	500 mg/twice a day curcumin capsule (95% turmeric root extract, containing 475 mg of cur- cuminoid) and 500 mg of calcium + 400 units of vitamin D daily (*n* = 26)	Placebo twice a day (*n* = 28)	8 weeks	Stomachache, hypertension (mild to moderate)	In the intervention group; the average serum MDA levels [MD (95% CI): −0.5 nmol/mL (−0.8 to −0.1); *p* = 0.009] and the hs-CRP [MD (95% CI): −0.5 mg/L (−0.8 to −0.2); *p* = 0.025] declined while serum TAC levels rose [MD (95% CI): 0.2 mmol/L (0.1 to 0.35); *p* < 0.001] There were no discernible variations in the serum biomarkers across the groups (MDA, TAC, and hs-CRP) There was no significant difference between the mean scores of the Greene scale, of the curcumin and placebo groups.
Kheiridoost et al., 2022 [44]	RCT-Triple-blind	Postmenopausal women who have been diagnosed with primary osteoporosis or osteopenia, mean age of control group (58.43 ± 3.41), mean age of Intervention 1 group (58.00 ± 3.50), and the mean age of Intervention 2 group (57.43 ± 3.80) (*n* = 120)	Intervention 1: 80 mg/day curcumin (nanomicelle capsule) (*n* = 30) Intervention 2: 80 mg curcumin (nanomicelle capsule) + 1000 mg *Nigella Sativa* oil (*n* = 28) All participants received one alendronate tablet (70 mg) weekly, and 500 mg calcium and 400 units of vitamin D daily.	Placebo (*n* = 29)	6 months	Severe belching (in Intervention 2)	There was a significant change in serum alkaline phosphatase levels in the Intervention 2 group (mean difference (MD) = −30.90, 95% CI: −50.28 to −11.53, *p* = 0.003). Also, a significant decrease in the mean differences of serum alkaline phosphatase levels in Intervention 2, compared to placebo, was observed (MD = −36.67, 95% CI: −66.19 to −7.15, *p* = 0.015). After adjusting for baseline values, there was a significant decline of serum alkaline phosphatase level in the placebo group compared to the Intervention 1 group (MD = −26.77, 95% CI: −52.22 to –1.33, *p* = 0.039) and the Intervention 2 group (MD = −36.74, 95% CI: −61.14 to –12.33, *p* = 0.004), and this difference was statistically significant. There was a significant decrease in serum OCN levels in Intervention 1 group (MD = −3.89, 95% CI: −7.41 to −0.37, *p* = 0.031). There was a significant decrease in serum OPN levels in the Intervention 1 group (MD = −6.50, 95% CI: −9.70 to −3.30, *p* < 0.001) and in the Intervention 2 group. (MD = −5.04, 95% CI: −10.79 to 0.72, *p* = 0.004). There were no significant differences in serum OCN and OPN levels between groups. Creatinine and alanine amino transferase (ALT) levels declined significantly throughout the research period in every group. There were no significant differences in serum ALT, aspartate amino transferase (AST), creatinine, and urea levels between groups.
Ahmed Nasef et al., 2022 [45]	RCT-Double blind, cross over	Postmenopausal women, median age = 58 (IQR, 55 to 61), (*n* = 16)	125 g/day bioactive coconut cream yoghurt (0.32 mg chlorogenic acid and 103 mg curcumin C3) (*n* = 16)	Placebo yoghurt (*n* = 16)	First trial, minimum one week wash-out, Second trial	-	Following their consumption of the bioactive yogurt, the intervention group’s plasma TNFα C_max_ was significantly lower compared to placebo (MD = 0.3 pg/mL; *p* = 0.04). Following consumption of the bioactive yogurt, there was a significant decrease in plasma TNFα from baseline, postprandially, while no differences were observed for the placebo. There were no differences between the intervention and placebo groups in terms of metabolic markers (triglycerides, insulin, and glucose).
Yousefi-Nodeh et al., 2024 [46]	RCT-Triple-blind	Postmenopausal women, 53.1% of the population, were 51–54 years old (*n* = 84)	500 mg/twice a day curcumin capsule (95% turmeric root extract, containing 475 mg of cur- cuminoid) (*n* = 26)	Placebo twice a day (*n* = 28)	8 weeks	Vomiting	The Curcumin-intervention group had significantly lower FBS (adjusted mean difference (MD) = −9.8, 95% CI: −16.7 to −2.8, *p* = 0.007), total cholesterol (MD = −26.0, 95% CI: −46.7 to −5.2, *p* = 0.015), and triglyceride levels (MD = −17.3, 95% CI: −29.7 to −5.0, *p* = 0.007) compared to the placebo. The mean serum levels of FBS (MD = −8.9, 95% CI: −14.9 to −2.9, *p* = 0.006), TC (MD = −21.3, 95% CI: −39.4 to −3.2, *p* = 0.023), LDL-C (MD = −24.5, 95% CI: −39.7 to −9.4, *p* = 0.003), and TG (MD = −13.4, 95% CI: −22.9 to −3.9, *p* = 0.008) significantly decreased from before to after the intervention. No significant differences were found in LDL-C and HDL-C, serum urea, creatinine, ALT, and AST levels. No significant decrease was found in the number of hot flashes among the curcumin group versus the placebo group. There was a significant decrease (MD = −13.2, 95% CI: −20.0 to −6.5; *p* < 0.001) in the number of hot flashes from before to after the intervention.
Farshbaf-Khalili et al., 2021 [47]	RCT-Triple-blind	Postmenopausal women with reduced bone mass density, 56–60 years old (47.5%), (*n* = 120)	Intervention 1: 80 mg/day curcumin (nanomicelle capsule) (*n* = 30) Intervention 2: 80 mg curcumin (nanomicelle capsule) + 1000 mg *Nigella sativa* oil (*n* = 30) All osteoporotic women received 70 mg of alendronate weekly, and all women supplemented with 500 mg of calcium + 400 units of vitamin D daily	Placebo (*n* = 30)	6 months	Nausea, belching (one person in Intervention 2 had severe), headache (one person in Intervention 1 had severe), and unpleasant taste for both intervention groups	There was a significant increase in miRNA-21 expression level in Intervention 2 (*p* = 0.043) from before to after the intervention. No significant differences were found between the groups in expression levels, of miRNA-422 and miRNA-503 before and after the intervention.
Ataei-Almanghadim et al., 2020 [48]	RCT-Triple-blind	Postmenopausal women, mean age 51.6 ± 5.4 (*n* = 89)	500 mg/twice a day curcumin oral capsule (*n* = 30)	Placebo twice a day (*n* = 30)	8 weeks	Diarrhea, headache, stomach ache, and vaginal bleeding	The mean number of hot flashes in the intervention group was significantly lower than that in the placebo group (MD = −10.7; 95% CI = −17.9 to −3.6; *p* = 0.001). No significant difference was found between the scores of Spielberger State-Trait Anxiety Inventory, the Female Sexual Function Index, and the Greene Scale of the groups.
Khanizadeh et al., 2018 [49]	RCT-Double blind	Postmenopausal women diagnosed with osteoporosis, mean age of control group (58 ± 12.45), mean age of alendronate + curcumin treated group (58 ± 10.78) (*n* = 60)	Alendronate (5 mg/day) + curcumin (110 mg/day) (*n* = 20) All participants received calcium supplements (1000–1500 mg/day) of calcium carbonate	Control (*n* = 20)	12 months	-	The bone-specific alkaline phosphatase level declined from the initial value in the Intervention group (13.16 ± 1.3 μg/L, 95% CI: 10.05–14.6, versus 10.2 ± 1.2 μg/L, 95% CI: 9.02–12.4, *p* = 0.012). At the end of the trial, the Intervention group’s BALP level was substantially different than that of the control group (*p* = 0.035). There was no significant difference in OCN levels. There was a significant decline in the levels of CTx in the alendronate + curcumin group, at the end of the study, compared to the initial (0.301 ± 0.017 μg/L, 95% CI: 0.27–0.42 versus 0.318 ± 0.033 μg/L, 95% CI: 0.23–0.35, *p* = 0.013). In the Intervention group, the mean serum level of CTx was significantly lower than the control group (*p* = 0.037). The mean hip bone mineral density of the intervention group significantly increased at the end of the study, compared to baseline values (0.829 ± 0.002, 95% CI: 0.76–0.84 versus 0.812 ± 0.006, 95% CI: 0.75–0.83, *p* = 0.01). In the intervention group, the total hip bone mineral density significantly increased compared to the control group at the end of the study (*p* = 0.046). In the Intervention group, there was an increase in the femoral neck bone mineral density levels at the end of the research compared to the initial (0.718 ± 0.001, 95% CI: 0.67–0.78 vs. 0.715 ± 0.002, 95% CI: 0.63–0.75, *p* = 0.063). The changes in the femoral neck bone mineral density in the intervention group compared to the control group were statistically significant (*p* = 0.030). In the Intervention group, the mean total body bone mineral density increased significantly at the end of the research compared to the initial measurement (1.005 ± 0.002, 95% CI: 0.98–1.45 vs. 0.981 ± 0.003, 95% CI: 0.93–1.33, *p* = 0.020). In the intervention group, the mean total body bone mineral density was significantly higher than the control (*p* = 0.014).

ALT: Alanine transaminase, AST: Aspartate transaminase, CI: Confidence interval, CTx: C-terminal cross-linking telopeptide of type I collagen, FBS: fasting blood sugar, FI: Fasting insulin, HDL: high-density lipoprotein, HDL-C: High-density lipoprotein cholesterol, HOMA-IR: Homeostatic Model Assessment for Insulin Resistance, hs-CRP: High sensitivity C-reactive Protein, IL-6: Interleukin-6, IQR: Interquartile range, LDL: low-density lipoprotein, LDL-C: Low-density lipoprotein cholesterol, MDA: Malondialdehyde, RCT: Randomized Controlled Trial, SOD: Superoxide dismutase, TAC: Total antioxidant capacity, TC: Total cholesterol, TNFα C_max_: Tumor necrosis factor-alpha maximum concentration, TNFα: Tumor necrosis factor-alpha.

**Table 3:** JBI Critical Appraisal Assessment For Randomized Controlled Trials.

Author, Year, Country	Q1	Q2	Q3	Q4	Q5	Q6	Q7	Q8	Q9	Q10	Q11	Q12	Q13	Total
Usefian et al., 2024 [39]	Y	Y	Y	Y	Y	Y	Y	N	U	Y	Y	Y	Y	11/13
Lustberg et al., 2024 [40]	Y	Y	N	Y	Y	N	Y	N	U	Y	Y	Y	Y	9/13
Salekzamani et al., 2023 [41]	Y	Y	Y	Y	Y	Y	Y	N	U	Y	Y	Y	Y	11/13
Sadeghzadeh et al., 2023 [25]	Y	Y	N	Y	Y	Y	Y	N	U	Y	Y	Y	Y	10/13
Iranshahi et al., 2023 [42]	Y	Y	N	Y	Y	Y	Y	N	U	Y	Y	Y	Y	10/13
Farshbaf-Khalili et al., 2022 [43]	Y	Y	Y	Y	Y	Y	Y	N	U	Y	Y	Y	Y	11/13
Kheiridoost et al., 2022 [44]	Y	Y	N	Y	Y	Y	Y	N	U	Y	Y	Y	Y	10/13
Ahmed Nasef et al., 2022 [45]	Y	Y	Y	Y	Y	U	Y	N	U	Y	Y	Y	Y	10/13
Yousefi-Nodeh et al., 2024 [46]	Y	Y	Y	Y	Y	Y	Y	N	U	Y	Y	Y	Y	11/13
Farshbaf-Khalili et al., 2021 [47]	Y	Y	N	Y	Y	Y	Y	N	U	Y	Y	Y	Y	10/13
Ataei-Almanghadim et al., 2020 [48]	Y	Y	Y	Y	Y	Y	Y	N	U	Y	Y	Y	Y	11/13
Khanizadeh et al., 2018 [49]	Y	U	Y	Y	Y	U	N	N	U	Y	Y	Y	Y	8/13

Q: Question, N: No, Y: Yes, U: Unclear.

## Data Availability

No new data were created or analyzed in this study. Data sharing is not applicable to this article.
